# Construction of an Efficient Genetic Transformation System for Watercress (*Nasturtium officinale* W. T. Aiton)

**DOI:** 10.3390/plants12244149

**Published:** 2023-12-13

**Authors:** Jiajun Ran, Qiang Ding, Yunlou Shen, Zhanyuan Gao, Guangpeng Wang, Yue Gao, Xiaoqing Ma, Xilin Hou

**Affiliations:** State Key Laboratory of Crop Genetics & Germplasm Enhancement, Key Laboratory of Biology and Genetic Improvement of Horticultural Crops (East China), Ministry of Agriculture and Rural Affairs of China, Engineering Research Center of Germplasm Enhancement and Utilization of Horticultural Crops, Ministry of Education of China, Nanjing Agricultural University, Nanjing 210095, China; 2020104055@stu.njau.edu.cn (J.R.); 2019204024@njau.edu.cn (Q.D.); 2020204027@njau.edu.cn (Y.S.); 2021204025@stu.njau.edu.cn (Z.G.); 2022204055@stu.njau.edu.cn (G.W.); 2021804158@njau.edu.cn (Y.G.); 2018204021@njau.cn (X.M.)

**Keywords:** *DR5* gene, transgenic watercress, auxin, efficient genetic transformation system

## Abstract

Based on the established efficient regeneration system for watercress in our laboratory, we optimized the processes of pretreatment, co-culture, and differentiation culture. Through GFP fluorescence and PCR identification, we successfully obtained transgenic watercress with the *DR5* gene, which allowed us to investigate the distribution details of auxin in the growth process of watercress. Our findings provide an effective method for gene function research and lay the foundation for innovative utilization of germplasm resources of watercress.

## 1. Introduction

Watercress (*Nasturtium officinale* W. T. Aiton.) is a widely cultivated aquatic vegetable that thrives in freshwater streams and mountain springs. Its natural growth is characterized by a robust root system and the ability to rapidly colonize available space through creeping vines and lateral branching. Watercress is rich in vitamins, minerals, flavonoids, and glucosinolates and has high nutritional value. Compounds extracted from this plant can be used for diuresis, hypoglycemia, neuroprotection, and anti-neuritis [1,2]. In addition, it is found that watercress has a certain ability to absorb heavy metal cadmium, which can alleviate cadmium pollution in the environment [3]. However, due to its aggressive growth, watercress has been classified as an invasive plant by certain countries [4]. Despite its innate vigor, watercress faces challenges in achieving efficient breeding and genetic manipulation. The absence of an effective regeneration system hinders the application of modern biotechnological approaches such as genetic transformation, asexual variation, and cell engineering in watercress breeding. Consequently, the identification and validation of desirable genes in watercress becomes a daunting task. Establishing a reliable and efficient regeneration system is therefore of paramount importance for watercress to achieve germplasm resource innovation. Moreover, it serves as a crucial pathway to expand market opportunities and achieve large-scale production of this valuable aquatic vegetable.

In this study, we aimed to investigate the root hair development mechanism in watercress and explore whether it is similar to that in Arabidopsis and other plants. We also aimed to develop watercress varieties with well-developed root systems that can adapt to land cultivation. Root hairs are crucial for water purification and nutrient absorption in watercress [5,6,7,8]. They develop from the differentiation of the root epidermis into H cells and are responsible for absorbing soil water and mineral elements and interacting with rhizosphere microorganisms [9]. Under unfavorable conditions such as drought or phosphorus deficiency, plants increase the production of root hairs to enhance water and phosphorus absorption and alleviate these conditions [10]. Auxin is known to be a direct influencing factor in plant root development, including root hair formation [11,12,13]. It regulates the development of the endodermis, root elongation, lateral root formation, and root hair formation. However, the mechanism of root hair formation in watercress and its similarity to other plants, such as Arabidopsis, is unknown. Watercress is commonly cultivated using hydroponics, but land cultivation is also possible. Expanding land cultivation of watercress is important for increasing the production scale [14]. Root activity is very important to plant growth and development. As an important part of the root system, root hair can not only increase the absorption area of the root system but also enhance the ability of the root system to absorb water and nutrients. They also interact with rhizosphere microbial communities to regulate the root environment [6]. The root is an important organ for watercress to adapt to dry land environments, purify water bodies, and increase yield. Watercress is mainly cultivated in water, which greatly limits the production and promotion of watercress. Watercress varieties with more developed roots can be obtained with genetic transformation, which is of great significance for watercress cultivation on land and is beneficial for watercress to gradually get rid of its dependence on hydroponics.

In conclusion, this study aimed to investigate the root hair development mechanism in watercress and develop watercress varieties suitable for land cultivation. Understanding the root hair formation process in watercress and its similarity to other plants will contribute to the development of improved varieties and increase production scale.

## 2. Materials and Methods

### 2.1. Materials

Watercress (serial number: DBC-001) came from the seed bank of the Chinese cabbage system biology laboratory of Nanjing Agricultural University, Nanjing, China.

*Agrobacterium tumefaciens* and related plasmids were provided by Nanjing Agricultural University.

### 2.2. Chemical Reagents

6-BA: 6-Benzylaminopurine (from Nanjing Zhengyang Biotechnology Co., Ltd., Nanjing, China); TDZ: Thidiazuron (from Nanjing Zhengyang Biotechnology Co., Ltd.); 2,4-D: 2,4-Dichlorophenoxyacetic acid (from Nanjing Zhengyang Biotechnology Co., Ltd.); NAA: Naphthaleneacetic acid (from Nanjing Zhengyang Biotechnology Co., Ltd.); KT: 6-Furfurylaminopurine (from Nanjing Zhengyang Biotechnology Co., Ltd.); ZT: Zeatin (from Nanjing Zhengyang Biotechnology Co., Ltd.); Cef: Cefalexin (from Nanjing Zhengyang Biotechnology Co., Ltd.); TMT: Timentin (from Nanjing Zhengyang Biotechnology Co., Ltd.); Kan: kanamycin (from Nanjing Zhengyang Biotechnology Co., Ltd.); AS: 3′,5′-Dimethoxy-4′-hydroxyacetophenone (from Nanjing Zhengyang Biotechnology Co., Ltd.).

### 2.3. Construction of DR5::EGFP Vector

**Figure 1:**
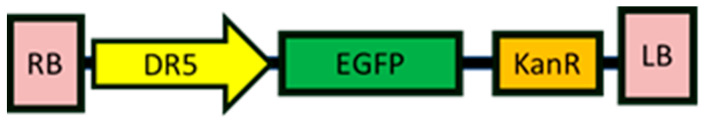
*DR5::EGFP* vector. *DR5**:*** an artificial DNA sequence, auxin response element. The *DR5* promoter contains many binding sites of ARF transcription factors which can respond to auxin signals to induce the expression of neighboring genes. The activity of *DR5* depends not only on the distribution of endogenous auxin but also on the signal of auxin. The plant response to auxin can be visualized by expressing appropriate reporter genes under the control of *DR5*.

To better understand the distribution of auxin in the growing points of watercress, we used the *DR5::EGFP* plasmid that had been constructed previously (Figure 1). The *DR5* promoter was cloned and inserted in front of the green fluorescent protein (GFP), and the expression of the GFP gene was induced by the *DR5* promoter.

### 2.4. PCR Positive Detection

PCR detection was performed using primers designed for EGFP with SnapGene 2.8.3. See Table S1 for details of the primers and procedures.

### 2.5. Observation of GFP Fluorescence

The regenerated watercress was placed under a stereomicroscope equipped with a GFP filter. The γ value was adjusted to 2, and the exposure time was set to 6 s. The samples were observed and photographed under a bright field and GFP fluorescence (see Figure S1 for details).

### 2.6. Efficient Regeneration of Watercress

The efficient regeneration system and hormone ratios for watercress are described in Table S2.

### 2.7. Mannitol Concentration and Treatment of Negative Pressure Time

The experimental results of the mannitol concentration and negative pressure time required for the pretreatment of the watercress genetic transformation system are detailed in Table S5.

### 2.8. Transformation Steps

(1) Select healthy watercress with consistent growth, remove the leaves, and cut the stem into sections of about 0.5 cm.
(2) Place the stem in the Murashige and Skoog (MS) medium (pH 5.2, supplemented with 0.6 M mannitol + 4 mg/L 6-Benzylaminopurine (6-BA)) for 24 h of pretreatment.
(3) Prepare the bacterial solution in lysogeny broth (LB) medium with antibiotics and activate it 24 h in advance. Two hours before infection, resuspend the bacteria in MS liquid medium (pH 5.2, containing 50 mg/L 3′,5′-Dimethoxy-4′-hydroxyacetophenone (AS), and remove phosphates). Then, activate the bacterial solution at 24 °C and adjust the optical density (OD) to 0.2~0.3 during infection.
(4) Wash the pretreated stem with sterile water, place it in a syringe, add the bacterial solution, and seal it for 3 min of vacuum infiltration.
(5) Inoculate the stem into the MS medium (pH 5.2, containing 4 mg/L 6-BA + 1.5 mg/L Thidiazuron (TDZ) + 1.5 mg/L 2,4-Dichlorophenoxyacetic acid (2,4-D) + 50 mg/L inositol + 10 mg/L AS), and cultivate in the dark at 24 °C for 3 days.
(6) Wash the stem with sterile water (containing 200 mg/L cefotaxime sodium (Cef) and 100 mg/L Timentin (TMT)), dry it, and place it in the MS medium (pH 5.2, containing 4 mg/L 6-BA + 1.5 mg/L TDZ + 1.5 mg/L 2,4-D + 50 mg/L inositol + 200 mg/L Cef + 100 mg/L TMT). Induce callus formation and observe GFP fluorescence by cultivating under light for 16 h a day and at 24 °C for 15 days.
(7) Place the callus on MS medium (pH 5.2, supplemented with 3 mg/L 6-BA + 3 mg/L TDZ + 200 mg/L Kanamycin (Kan)), cultivate under light for 16 h a day at 24 °C for 15 days, and induce the differentiation of large amounts of resistant buds.
(8) Inoculate the resistant buds into the MS medium (pH 5.7, containing 200 mg/L Kan), cultivate under light for 16 h a day at 24 °C for 7 days, induce root formation, and observe. Before transplanting the seedlings, the cap of the tissue culture bottle was half-opened and half-closed 3 days in advance, and the regenerated plants of watercress were domesticated in the light culture box, and then, the seedlings were removed from the tissue culture bottle.
(9) The regenerated plants were adapted to tissue culture in vitro (it was cultured in water with Hogan’s solution and cultured at 24 °C for 16 h), and PCR detection was performed.

### 2.9. Statistical Analysis

All of the data are presented as the means and standard deviation (SD) of at least three independent replicates for each experiment. Student’s two-tailed t-test was conducted using GraphPad Prism version 8.0 (GraphPad Software, San Diego, CA, USA).

## 3. Results

### 3.1. Effects of Common Hormones on Stem Segments of Watercress

**Figure 2:**
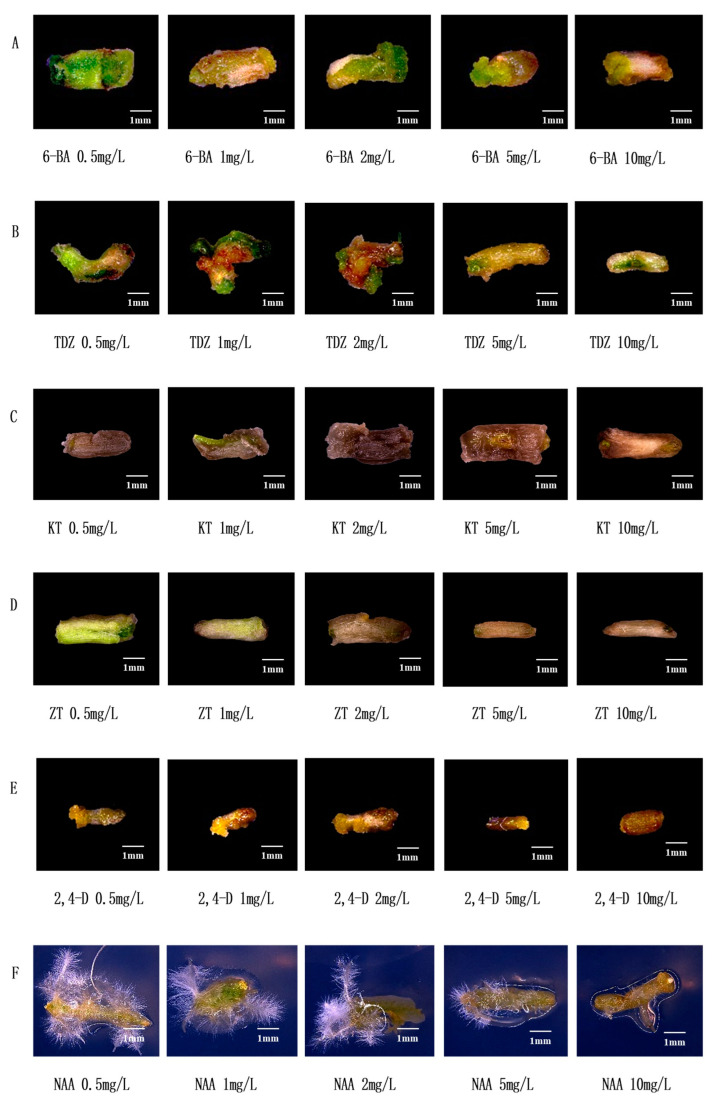
Effect of common plant hormones on stem segments of watercress. (**A**) Effect of 6-BA on stem segments of watercress. (**B**) Effect of TDZ on stem segments of watercress. (**C**) Effect of KT on stem segments of watercress. (**D**) Effect of ZT on stem segments of watercress. (**E**) Effect of 2,4-D on stem segments of watercress. (**F**) Effect of NAA on stem segments of watercress.

6-BA is one of the commonly used cytokinins which can induce callus formation of watercress (Figure 2A). When the concentration of 6-BA was 0.5 mg/L, the stem segment and callus were green, and the callus was smaller; when the concentration of 6-BA increased gradually, the stem segment gradually ruptured and the callus became larger; when 6-BA ≥ 5 mg/L, the stem segment browned, and the callus volume became smaller. Therefore, the suitable concentration range of 6-BA is 2~5 mg/L. When the concentration of TDZ is 0.5 mg/L, the stem segment bends, the callus is small, and the color is white; when the concentration of TDZ is 1 mg/L, the stem segment browns, and the callus is still small; when the TDZ concentration is 2 mg/L, the stem segment breaks, browns, and the callus is small and green; when the TDZ concentration ≥ 5 mg/L, the stem segment color turns white and does not form calli (Figure 2B). Therefore, the suitable concentration range of TDZ is 1~2 mg/L. KT is one of the commonly used cytokinins. Figure 2C shows that KT has a toxic effect on the stem segment of watercress, resulting in serious browning, which is not conducive to callus formation. The effect of ZT on watercress is similar to that of KT (Figure 2D) but not suitable for tissue culture of watercress. 2,4-D is one of the commonly used cell auxins which can induce the callus of watercress (Figure 2E). With the increase in the concentration of 2,4-D, the stem segment browns and the callus becomes larger; when 2,4-D ≥ 5 mg/L, the stem segment browns and does not form calli. Therefore, the suitable concentration range of 2,4-D is 1~2 mg/L. NAA is one of the plant auxins which can induce the rooting of watercress (Figure 2F). When the concentration of NAA was 0.5~2 mg/L, the stem segment was curved and yellowish green, and a large number of roots sprouted; when the concentration of NAA was greater than or equal to 5 mg/L, the stem segment was yellow, sprouting a little or not sprouting roots. Therefore, the NAA of 0.5~1 mg/L is suitable for tissue culture of watercress.

### 3.2. Establishment and Optimization of Regeneration System

When the stem segments of watercress were inoculated in the MS medium for 15 days and when the concentrations of 6-BA and TDZ were constant, the callus rate decreased gradually with the increase in 2,4-D concentration. The callus rate increased with the increase in TDZ when the concentrations of 6-BA and 2,4-D were constant. The callus rate increased with the increase in 6-BA concentration when the concentrations of TDZ and 2,4-D were constant. Finally, we found that 6-BA at the concentration of 4 mg/L, TDZ of 1.5 mg/L, and 2,4-D of 1.5 mg/L had the highest efficiency of inducing calli from stem segments (Table 1).

When the callus was cultured on MS medium for 10 days and when the concentration of 6-BA was 2~3 mg/L, the number of adventitious buds increased with the increase in TDZ concentration, and when the concentration of 6-BA was 5 mg/L, the number of adventitious buds decreased with the increase in TDZ concentration. We finally decided to use the concentration of 3 mg/L 6-BA and TDZ to induce watercress calli to differentiate adventitious buds, and this combination had the highest efficiency in inducing calli to produce adventitious buds (Table 2).

When the adventitious buds were inoculated into the MS medium for 10 days, we found that the adventitious buds of watercress could take root even without NAA in the medium. When the concentration of NAA in the medium was 0.3~0.7 mg/L, adventitious buds could be induced to take root, but the root length would be shorter (Table 3). Therefore, we suggest that MS medium be directly used to induce the rooting of adventitious buds of watercress.

**Figure 3:**
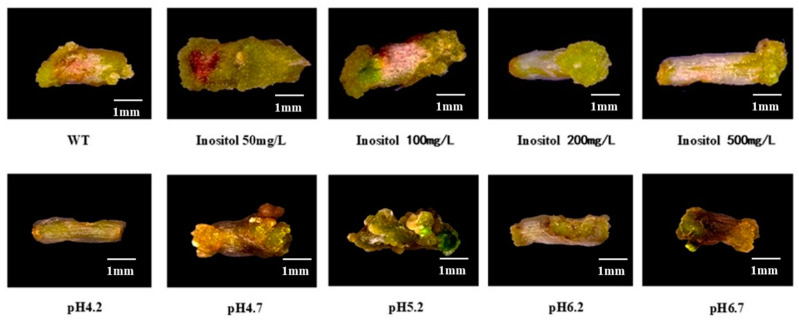
Effects of different inositol concentrations and pH on callus formation of watercress.

Inositol and culture medium pH had obvious effects on plant tissue dedifferentiation and callus formation. We took the regeneration system as control and observed the stem segment of watercress by adding different inositol concentrations and setting different pH. Figure 3 shows that the addition of 50 mg/L inositol to the original regeneration system can promote callus expansion. When the pH was close to 5.2, the number of calli formed at the wound of the stem segment was significantly higher. Therefore, adding 50 mg/L inositol to the regeneration system and adjusting pH to 5.2 can form a more efficient regeneration system.

**Figure 4:**
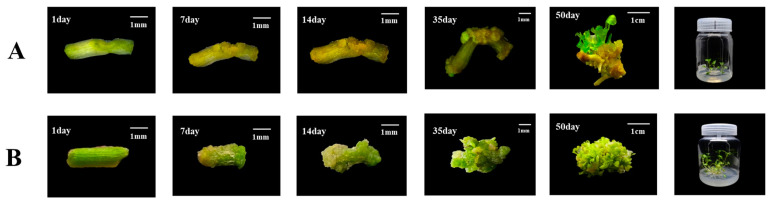
Comparison of watercress regeneration system before (**A**) and after (**B**) optimization.

In summary, the stem segment of watercress was used as material to induce calli by adding 6-BA 4 mg/L + TDZ 1.5 mg/L + 2,4-D 1.5 mg/L + inositol 50 mg/L to the MS medium of pH 5.2, 6-BA 3 mg/L + TDZ 3 mg/L was added to the MS medium of pH 5.2 to induce callus differentiation and bud differentiation, and MS medium was used to induce adventitious bud rooting. Finally, an efficient regeneration system of watercress with a period of about 50 days was constructed (Figure 4).

### 3.3. Genetic Transformation Steps

**Figure 5:**
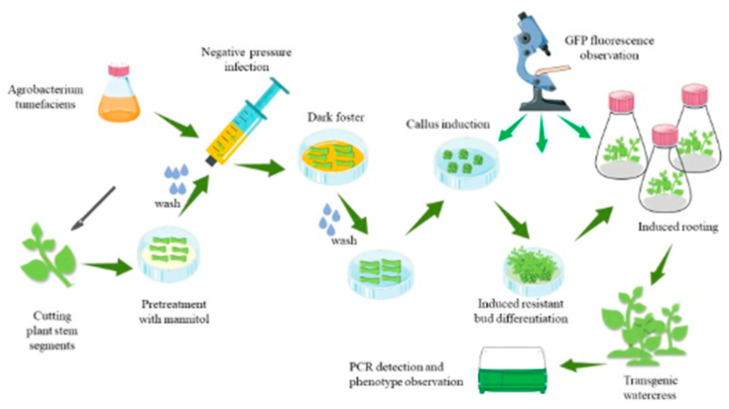
Steps of the genetic transformation system of watercress.

As shown in Figure 5, the first step is to prepare *A. tumefaciens* and stem segments of the watercress. The second step is to pretreat the stem segments, and the third step is to infect the stem segments with an injection syringe for 3 min. The fourth step involves co-cultivation of the stem segments with *A. tumefaciens* in darkness. The fifth step is to induce callus formation by washing the stem segments and placing them in a specific medium. The sixth step is to induce the callus to form resistance buds. The seventh step involves the induction of resistant buds to form roots, and the eighth step involves the cultivation of plants with resistance through domestication and planting.

**Figure 6:**
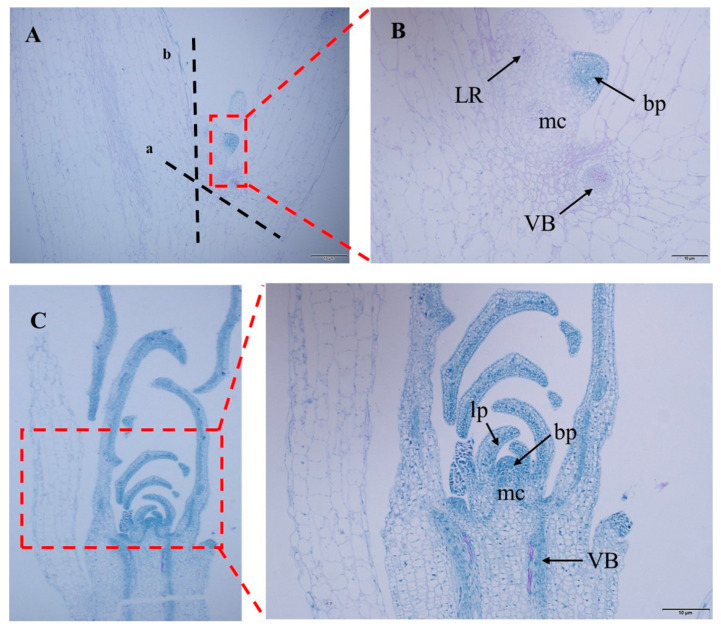
Meristem tissue slice of watercress. (**A**) Watercress leaf axil slice. (**B**) Enlarged view of leaf axil slice. (**C**) Slice of growth points of watercress. ab—cut point of watercress stem section. LR—root primordium. mc—meristematic cell. bp—bud primordium. lp—leaf primordium. VB—vascular bundle.

The specific operations are as follows: Cut off the bud primordia at the stem–leaf axil according to the ab dashed line in Figure 6A. After the stem is cut, preculture it in the MS medium (containing 0.6 M mannitol) for 24 h in the dark. Wash the pretreated stem segment, and place it together with the bacterial solution in the syringe. Draw in twice the volume of the stem segment of the bacterial solution, exclude the excess gas in the syringe, remove the needle, seal it with flames to form a closed environment, and pull the piston to form a negative pressure environment. This process lasts for about 3 min, shaking constantly to remove gas from the stem. If the time is too long, the stem will become transparent. If the time is too short, the bacterial solution cannot fully be in contact with the wound. After the infection, suck up the residual solution and place the stem in the co-culture medium, and co-culture in the dark at 24 °C for 3 days. Rinse the stem after the co-culture with sterile water for 10–20 min. The rinsed stem is then inoculated into the callus induction medium and incubated at 24 °C under 16 h of light for 15 days. During this process, the callus will gradually form and enlarge. The enlarged callus is then inoculated into the shoot differentiation medium and incubated at 24 °C under 16 h of light for 15 days. The callus will form resistant buds after Kan screening. Cut the appropriate size of resistant buds and inoculate them into the MS culture medium. Incubate at 24 °C under 16 h of light for 7 days, and the buds will spontaneously form roots. After the roots grow into the MS culture medium for 10 days, the regenerated seedlings are domesticated and transplanted.

### 3.4. Identification of Transgenic Watercress

**Figure 7:**
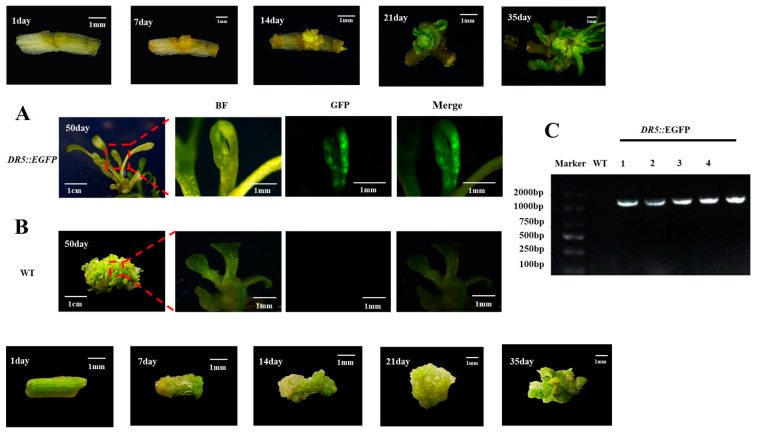
Regeneration process and PCR and GFP detection of the watercress. (**A**) Regeneration and GFP detection results of *DR5::EGFP* watercress. (**B**) Regeneration and GFP detection results of WT watercress. (**C**) PCR identification results of *DR5::EGFP* watercress.

We conducted a tracking study of the genetic transformation system of watercress (as shown in Figure 7A,B). During the 50-day regeneration process, we recorded the development of the control group and *DR5::EGFP* transgenic watercress and conducted GFP fluorescence and PCR detection. The transformation efficiency could reach up to 10.6% (Table S4). The color of the watercress stem segment changed from green to yellow after pretreatment. After 7–14 days of induction of calli, the control group formed a large number of yellow-green calli at the wound site, while the *DR5::EGFP* transgenic watercress formed yellow calli with stronger granules. After 21–35 days of growth on the shoot differentiation medium, the control group’s callus continued to grow for a period of time before differentiating into a large number of green shoot buds. The *DR5::EGFP* transgenic watercress, on the other hand, differentiated into a small number of green-resistant shoot buds within a short period of time, and the buds continued to grow. At about 50 days, the control group of watercress formed a large number of shoots, while the number of regenerated shoots in *DR5::EGFP* transgenic watercress was rare.

After observing the GFP fluorescence of regenerated seedlings of the control group and *DR5::EGFP* transgenic watercress, it was found that the GFP fluorescence signal was mainly concentrated near the growing point of the regenerated seedlings. Therefore, the growing point of the watercress was made into paraffin sections (Figure 5). Combined with the GFP fluorescence distribution of *DR5::EGFP* transgenic watercress and the structure of the growing point, it was found that the main site of auxin in the growing point of watercress was in the bud primordium, leaf primordium, and meristematic zone, while there was no obvious GFP fluorescence signal in other areas. This indirectly indicates that during the growth process of watercress, the lateral branches of watercress can germinate, and the polarity distribution of auxin near the growing point is closely related.

## 4. Discussion

Watercress, a valuable source of nutrients with high nutritional and medicinal value, has garnered significant research attention in areas such as pharmacology, cultivation methods, and water purification [13,15]. However, studies focusing on the molecular mechanism and cell engineering breeding of watercress remain scarce. Building upon our exploration of the effects of common cytokinins and auxins on stem segments, we conducted gradient experiments to establish a more efficient regeneration system with a cycle of 50 days. Further optimization was achieved through inositol and pH gradients, ultimately leading to the development of a highly efficient regeneration system. Our work successfully bridges the gap in efficient watercress regeneration systems, both domestically and internationally.

Transcriptome sequencing analysis of watercress has revealed numerous candidate genes related to metabolism, such as phenylpropanoid biosynthesis and glucosinolate metabolism [16,17,18]. Through sequence alignment, many genes related to phosphorus starvation response, roots, and root hair development were identified in watercress [19]. However, the lack of an effective genetic transformation system has hindered the functional validation of these candidate genes. The construction of a genetic transformation system faces many unknown challenges. Different plant hormones have varying effects on watercress regeneration. After conducting gradient experiments with a single hormone concentration, we selected the plant hormone that had the least harm to stem segments and the most significant effect to establish the watercress regeneration system (Table 1, Table 2 and Table 3). Inositol is often added to plant tissue culture to regulate cell division, which has a significant effect on callus development. The level of endogenous inositol in soybean decreased and cell division was inhibited. On the contrary, in the process of tissue culture, the concentration of inositol was on the high side, and the inhibition of cell division was not conducive to callus growth [20,21]. The addition of 50 mg/L of inositol to the watercress callus induction medium significantly increased the callus volume (Figure 3). Tissue sections of the watercress showed that the cell walls at the leaf axils were relatively thick compared to other parts (Figure 5), which hindered sufficient contact between the Agrobacterium and meristematic cells. Therefore, we used different concentrations of mannitol to determine that 0.6 M mannitol could dehydrate the protoplasts of watercress while keeping them alive (Table S5). Watercress is rich in glucosinolates and can inhibit the activity of fungi, which is one of the reasons why genetic transformation is difficult to succeed [22]. According to the report, the tissue of grape and other plants will isolate the bacterial liquid, and the negative pressure infection is beneficial to the direct contact between the bacterial liquid and the material and increases the probability of infection [23,24]. Thus, stem segments were pretreated with 0.6 M mannitol + 4 mg/L 6-BA before infection, allowing a large number of meristematic cells to undergo protoplast wall separation while remaining active. The purpose of using negative pressure infiltration in the syringe was to ensure sufficient contact between the bacterial solution and watercress cells without affecting the viability of the stem segments. During the optimization process of the regeneration system, it was found that a pH 5.2 culture environment could induce abundant callus formation. We believed that this was due to the effect of the lower pH on the form of plant hormones such as auxin (Table 1, Table 2 and Table 3). Moreover, an acidic environment is also beneficial for activating the vir genes. Therefore, the pH of the environment was maintained at 5.2 from infection to the differentiation of resistant buds. From the success rate of our genetic transformation, it can be seen that mannitol pretreatment is the key to the genetic transformation system, while negative pressure infection can only increase the transformation success rate. 

Currently, there are very few reports on genetic transformation methods in watercress, mainly through root-inducing Agrobacterium-mediated transformation to obtain transgenic watercress roots. Nam Il Park constructed a root-inducing Agrobacterium-mediated transformation method [25] and successfully obtained transgenic watercress roots with the reporter gene GUS [26]. However, there have been no reports on the genetic transformation of watercress shoots or the regeneration of whole transgenic watercress plants [27]. In our study, we successfully established a genetic transformation system for watercress shoots using Agrobacterium-mediated transformation. The key steps of this system include mannitol pretreatment of stem segments, negative pressure infiltration of the bacterial solution, and maintaining a pH 5.2 culture environment. By optimizing these steps, we were able to achieve high transformation efficiency and obtain transgenic watercress shoots. The transgenic watercress shoots obtained in our study were confirmed using PCR analysis and showed stable expression of the introduced gene. This provides a foundation for further functional studies and genetic improvement of watercress. In conclusion, we have developed a successful genetic transformation system for watercress shoots using Agrobacterium-mediated transformation. This system can be used for the introduction of desirable traits into watercress and for the study of gene functions in this important vegetable crop. Further research is needed to optimize the regeneration process and to explore the potential applications of this system in watercress breeding and genetic improvement.

## Figures and Tables

**Table 1:** The proportion of hormones needed to induce calli in stems of watercress.

Number	6-BA mg/L	TDZ mg/L	2,4-D mg/L	Number of Inoculated Stem Segments	Callus Rate (%)
CK	-	-	-	300	0.00 ± 0.00 ^a^
1-1	2	1	1.5	300	80.32 ± 1.53 ^b^
1-2	2	1	2	300	73.67 ± 2.08 ^c^
1-3	2	1	2.5	300	64.67 ± 1.53 ^d^
2-1	2	1.5	1.5	300	84.33 ± 3.05 ^b^
2-2	2	1.5	2	300	79.67 ± 1.53 ^c^
2-3	2	1.5	2.5	300	73.68 ± 2.08 ^d^
3-1	3	1	1.5	300	92.67 ± 1.46 ^b^
3-2	3	1	2	300	84.67 ± 1.45 ^c^
3-3	3	1	2.5	300	76.33 ± 2.52 ^d^
4-1	3	1.5	1.5	300	94.34 ± 1.53 ^b^
4-2	3	1.5	2	300	89.00 ± 2.65 ^c^
4-3	3	1.5	2.5	300	85.67 ± 3.07 ^d^
5-1	4	1	1.5	300	91.67 ± 0.58 ^b^
5-2	4	1	2	300	86.01 ± 1.00 ^c^
5-3	4	1	2.5	300	80.33 ± 1.53 ^d^
6-1	4	1.5	1.5	300	98.00 ± 2.00 ^b^
6-2	4	1.5	2	300	95.01 ± 1.00 ^c^
6-3	4	1.5	2.5	300	92.67 ± 2.08 ^d^

Note: “-” means not to add. Values indicate mean ± SD. Letters indicate significant difference analysis (*p* < 0.05, Student’s *t*-test).

**Table 2:** Hormone ratios for inducing adventitious buds from calli of watercress.

Number	6-BA mg/L	TDZ mg/L	2,4-D mg/L	NAA mg/L	Number of Inoculated Calli	Number of Buds
CK	-	-	-	-	300	0 ^a^
1	2	1	-	-	300	1422 ^b^
2	2	2	-	-	300	2267 ^c^
3	2	3	-	-	300	3697 ^d^
4	3	1	-	-	300	2788 ^e^
5	3	2	-	-	300	4059 ^f^
6	3	3	-	-	300	5111 ^g^
7	5	1	-	-	300	2453 ^h^
8	5	2	-	-	300	1930 ^i^
9	5	3	-	-	300	1151 ^j^

Note: “-” means not to add. Letters indicate significant difference analysis (*p* < 0.05, Student’s *t*-test).

**Table 3:** NAA concentration for inducing rooting of adventitious buds of watercress.

Number	NAA mg/L	Rooting Rate	Rooting Number (Root/Plant)	Root Length (cm)
CK	-	100%	21.67 ± 0.58 ^a^	6.30 ± 0.1 ^a^
1	0.3	100%	35.67 ± 0.58 ^b^	5.33 ± 0.25 ^b^
2	0.5	100%	30.00 ± 1 ^c^	3.90 ± 0.36 ^c^
3	0.7	100%	27.00 ± 1 ^d^	1.63 ± 0.15 ^d^

Note: “-” means not to add. Values indicate mean ± SD. Letters indicate significant difference analysis (*p* < 0.05, Student’s *t*-test).

## Data Availability

Data are available within the article and Supplementary Materials.

## Supplementary Materials

The following supporting information can be downloaded at: https://www.mdpi.com/article/10.3390/plants12244149/s1, Figure S1. *DR5: GFP* fluorescence of EGFP transgenic watercress; Table S1. PCR detection parameters; Table S2. Efficient regeneration parameters of watercress; Table S3. Formula of genetic transformation system of watercress; Table S4. Conversion rate of genetic transformation system of watercress; Table S5. Effect of different concentrations of mannitol on protoplast morphology of watercress.
