# Supplementary material for: Construction of an Efficient Genetic Transformation System for Watercress (Nasturtium officinale W. T. Aiton)
Source: Plants (Basel). 2023 Dec 13;12(24):4149. doi: 10.3390/plants12244149 (PMC10747978; doi:10.3390/plants12244149)
Supplement: Supplementary file 1 [file plants-12-04149-s001.zip › plants-2729554-supplementary.pdf]

Table S1. PCR detection parameters.

**Mixture Composition**	**Parameter**
EGFP-F	AGGGGATCCATGGCTCCAAAGAA
EGFP-R	CCATCTAATTCAACAAGAATTGGGACAAC
Template	1 μL
dNTP Mixture（2.5 mM each）	4 μL
10×LA PCR Buffer II（Mg^{2+} plus）	2.5 μL
TaKaRa LA Taq（5 U/μL）	0.25 μL
AP1 Primer（100 pmol/μL）	0.5 μL
SP1 Primer（10 pmol/μL）	0.5 μL
ddH2O	up to 25 μL
Total	25 μL

The reaction procedure is:
Denaturation at 94 °C for 30 s; 60 °C annealing for 1 min; Extend for 3 min at 72 °C; Denaturation at 94 °C for 30 s;60 °C annealing for 1 min; Extend for 3 min at 72 °C; Repeat 30 cycles; Extend for 10 min at 72 °C.

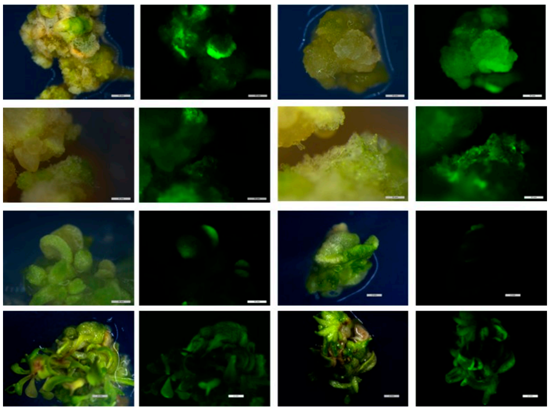

Figure S1. ***DR5: GFP*** **fluorescence of EGFP transgenic watercress.**

Table S2. Efficient regeneration parameters of watercress.

**Procedure Steps**	**Medium Type**	**pH**	**6-BA (mg/L)**	**TDZ (mg/L)**	**2,4-D (mg/L)**	**Inositol (mg/L)**
Callus induction	MS	5.2	4	1.5	1.5	5-
Induced differentiation bud	MS	5.2	3	3	-	-
Induced rooting	MS	5.7	-	-	-	-

Note: "-" means not to add

Table S3. Formula of genetic transformation system of watercress.

Procedure Steps	Medium Type	pH	6-BA mg/L	TDZ mg/L	2,4-D mg/L	AS mg/L	Inositol mg/L	Cef mg/L	TMT mg/L	Kna mg/L	Time	Matters Needing Attention
pretreatment	MS（l）	5.2	4	-	-	-	-	-	-	-	24h	Dark culture
Agrobacterium infection	MS（l,-Pi）	5.2	-	-	-	50		-	-	-	3mim	Negative pressure; OD value 0.2~0.3
coculture	MS（S）	5.2	4	1.5	1.5	10	50	-	-	-	3day	24°C ;dark environment culture
Callus induction	MS（S）	5.2	4	1.5	1.5	-	50	200	100	-	15day	24°C; Cultivation under 16h light
Induced differentiation bud	MS（S）	5.2	-	-	-	-	-	200	100	100	15day	24°C; Cultivation under 16h light
Induced rooting	MS（S）	5.7	4	1.5	1.5	-	50	200	200	100	14day	24°C; Cultivation under 16h light

Note: "-" means not to add

Table S4. Conversion rate of genetic transformation system of watercress.

Number	Handle		Stem Segment Phenotype	Conversion Rate
	0.6M Mannitol Pretreatment (h)	Negative Pressure Time (min)		
1	-	-	normal	-
2	-	2	normal	-
3	-	3	normal	-
4	-	5	vitrified	-
5	12	-	green	-
6	24	-	yellow-green	-
7	36	-	Reduced volume; Color yellow	-
8	48	-	Reduced volume; withered and yellow	-
9	12	3	normal	1.7%
1-	24	3	Slightly vitrified	3.6%

Note: "-" indicates the probability of not adding 0 or 0

Table S5. Effect of different concentrations of mannitol on protoplast morphology of watercress.

Number	Mannitol Concentration (M)	Time (h)	Protoplast State
1	0.1	12h	Imbibition rupture
2	0.2	12h	Imbibition rupture
3	0.3	12h	Imbibition rupture
4	0.4	12h	extending
5	0.5	12h	complete
6	0.6	12h	Slight dehydration and shrinkage
7	0.7	12h	Shrinkage due to dehydration
8	0.8	12h	Shrinkage due to dehydration
9	0.9	12h	Shrinkage due to dehydration
